# Climbable mountain

**DOI:** 10.1002/jhm.70064

**Published:** 2025-04-17

**Authors:** Nike Izmaylov, Michelle Izmaylov

**Affiliations:** ^1^ Vanderbilt University Medical Center Nashville Tennessee USA

There is a traveler on the road. The traveler has been through a long and difficult journey, and now the traveler arrives at a mountain stretching high up. Theoretically, the traveler could go around the mountain, but this would set the traveler back a few days. The traveler has companions, and the efficiency of the journey is of utmost significance to them.

The traveler decides to scale the mountain.

Climbing the mountain is treacherous and perilous. The traveler confronts many dangers, the crumble of rock beneath feet, and the peril of a narrow path along a cliff.

But the traveler gets to the summit.

The traveler considers the difficult journey that led here. They drop their backpack to the ground, the pain from the straps felt in their shoulders, the pain in their muscles was a reminder of the difficulty of the climb.

But the mountain *was* climbable. This challenge, though difficult, *was* surmountable.

Then, the traveler looks toward the road ahead. What they find: many more mountains.

The traveler hesitates. The road is much more challenging than anticipated.

But the traveler's companions are shocked by this hesitation. The traveler just demonstrated the capability to climb a challenging mountain. Why hesitate at the prospect of climbing all the other mountains?

The traveler considers how many supplies this climb demanded, how much effort, the exhaustion felt now at the summit.

The companions respond: but you *did* climb this mountain. This mountain *was* surmountable. Each of these other mountains is *also* surmountable. There is truth to this. *Each* mountain is surmountable. But are *all* mountains, consecutively, surmountable?

The traveler recognizes there is an alternative: to go around the other mountains, to have a journey with less challenge and more enjoyment. But the companions are expecting the traveler to pursue the most efficient route.

The traveler continues to climb the mountains instead of going around.

Each mountain seems more difficult. There is perhaps no difference from the perspective of objective terms, but the traveler feels the mounting challenge of each step. There's a fall, then a strain, each injury increasing the possibility of another error during the climb.

Then, the traveler reaches their limit. The injuries they have accumulated have rendered them not capable of climbing any more mountains, at least not without a significant opportunity to recover.

But there is a mountain ahead. The traveler recognizes that this is the mountain they must go around. They are too exhausted to climb. There is no alternative.

When the traveler approaches, they recognize there is no manner in which to go around this mountain. There are even more difficult mountains flanking this mountain, no other path but to climb. There is no alternative.

The traveler recognizes that, perhaps if they had not climbed all the mountains that they could have gone around, they could have gathered all their strength and climbed this one, singular mountain. But they have already expended all their strength. They do not have the strength to climb this mountain, they can't go around.

This mountain was not insurmountable at the start of the journey. But now, this mountain that could have been climbable is climbable no longer, and the whole journey ahead seems insurmountable.

This is just a parable.

But when I hear residents talking about having to admit a patient at the end of their shift, when I hear outpatient clinicians staying hours after their shifts to complete documentation, when I hear inpatient proceduralists continuing to do work even when they're exhausted—I think about the traveler. For each of these individual situations, it is feasible for the clinician to stay late or work excessively. These challenges are all individually surmountable.

But when we ask clinicians to do this repeatedly—when residents stay after signout each day, when piles of documentation accompany each outpatient clinic shift, when clinicians confront the demand of doing many procedures each day—we must consider whether such consecutive effort is surmountable.

Even when a clinician is able to climb each individual mountain, there arrives a moment when the clinician is not able to climb the next mountain to do what is needed for their patient. When pushed at such a moment—when the clinician perceives the system demands they climb more mountains when they can climb no longer—burnout and suicide might become the answer.

Instead of discussing what is *surmountable*, we need to discuss what is *sustainable*.

We can contribute to preventing burnout by having a culture of going around mountains, even at the cost of efficiency, and reserving the climb for when we must. There are moments when we must step up for colleagues and patients, moments when too many clinicians have their own challenges and can't be at their shifts, moments when too many patients are at the hospital without sufficient staff—but these should not be incidents that occur each day when we could instead plan for alternative routes.

When we ask clinicians to climb mountains each day for efficiency, we do not leave reserve for the moments when there is no alternative but to climb mountains to provide care for patients.

This demands a shift in the culture of the profession. In medicine, we pressure clinicians to do as much work as possible. We expect constant efficiency and continuous effort, demand that clinicians stay for hours after their shifts and provide patient care in an environment of intensity that makes many feel overwhelmed. We consider each incident a mountain that must be climbed. We can't continue to speak of each mountain as an isolated event. We can't continue to demand that clinicians do any work they are theoretically capable of doing regardless of the cost. When clinicians climb mountains each day, the work is no longer sustainable.

Systematic solutions are essential for many challenges in medicine. Individual clinicians can't increase staffing or decrease the patient census in their clinical setting. However, there is much we can do at the level of individual clinicians while we work toward those systematic solutions.

We can shift to a culture of finding approaches to go around mountains instead of expecting our colleagues to climb each mountain they encounter.

The resident expected to stay for hours to complete an admission received at the end of a shift—we could instead expect the resident to stabilize the patient and then sign out, letting the overnight service do a more thorough job with the admission during their shift hours. The nurse practitioner overwhelmed with too many consults—we can establish a process for offloading consults in that scenario, setting expectations to account for a work environment that is too demanding to provide excellent patient care. The attending struggling with too much documentation to complete—we can remind the attending that the purpose of documentation is to communicate, setting expectations to prioritize communication instead of billing codes when there's too much work to do during a shift.

Instead of pressuring our colleagues to climb as many mountains as possible, we can learn to be better traveling companions and find solutions that make work easier when possible.

When we look at a mountain, we should not think: can I climb this mountain at this moment?

When we look at a mountain, we should think: can I go *around* this mountain at this moment, reserving strength for the mountain that I *must* climb?

There are going to be mountains we must climb. But by making each mountain sustainable, we can make the whole journey surmountable.

## CONFLICT OF INTEREST STATEMENT

The authors declare no conflict of interest.

